# Improved Determination of Q Quality Factor and Resonance Frequency in Sensors Based on the Magnetoelastic Resonance Through the Fitting to Analytical Expressions

**DOI:** 10.3390/ma13214708

**Published:** 2020-10-22

**Authors:** Beatriz Sisniega, Jon Gutiérrez, Virginia Muto, Alfredo García-Arribas

**Affiliations:** 1 Departamento de Electricidad y Electrónica, Universidad del País Vasco/Euskal Herriko Unibertsitatea (UPV/EHU), Barrio Sarriena s/n, 48940 Leioa, Spain; jon.gutierrez@ehu.eus (J.G.); alfredo.garcia@ehu.es (A.G.-A.); 2 BCMaterials, Basque Center for Materials, Applications and Nanostructures, UPV/EHU Science Park, 48940 Leioa, Spain; 3 Departamento de Matemática Aplicada y Estadística e Investigación Operativa, Universidad del País Vasco UPV/EHU, P.O. Box 644, 48080 Bilbao, Spain; virginia.muto@ehu.eus

**Keywords:** magnetoelastic resonance, quality factor, resonance curve fit

## Abstract

The resonance quality factor Q is a key parameter that describes the performance of magnetoelastic sensors. Its value can be easily quantified from the width and the peak position of the resonance curve but, when the resonance signals are small, for instance when a lot of damping is present (low quality factor), this and other simple methods to determine this parameter are highly inaccurate. In these cases, numerical fittings of the resonance curves allow to accurately obtain the value of the quality factor. We present a study of the use of different expressions to numerically fit the resonance curves of a magnetoelastic sensor that is designed to monitor the precipitation reaction of calcium oxalate. The study compares the performance of both fittings and the equivalence of the parameters obtained in each of them. Through these numerical fittings, the evolution of the different parameters that define the resonance curve of these sensors is studied, and their accuracy in determining the quality factor is compared.

## 1. Introduction

Magnetoelastic resonance sensors are usually made of amorphous, ribbon-shaped ferromagnetic alloys [1]. Magnetostriction in these materials provides a strong coupling of their magnetic and mechanical properties, so that when an external alternating magnetic field is applied to a magnetoelastic ribbon, elastic waves are induced in it. Conversely, the magnetic state of these materials is highly dependent on the application of external mechanical forces and loads, and therefore these mechanical variations generate changes in the magnetic flux that can be remotely detected by a detection coil placed on the ribbon. Matching the physical dimensions and elastic properties of the material used in the sensor, the phenomenon of magnetoelastic resonance occurs at specific frequencies that can be expressed as:

(1) $$f_{n}=\frac{n}{2L}\sqrt{\frac{E}{\rho }}$$

where L is the length of the ribbon, E is the Young’s modulus of the material, ρ its density, and n=1 corresponds to the fundamental resonant frequency. This resonant behavior is highly sensitive to different external parameters, which has generated a great interest in the use of these materials as a main part of different sensing systems [2]. Magnetoelastic sensors have been used to measure several environmental parameters such as pressure, humidity or temperature [3,4], liquid viscosity and density [5,6], chemical agents and pH [7,8], and biological agents [9,10,11]. Specifically, and with respect to its use as a mass sensor, when the magnetoelastic ribbon is loaded with a uniform and small amount of mass (δm), a shift in its resonance frequency occurs, that can be evaluated from Equation (1) to be, to first order approximation:

(2) $$\delta f=-\frac{1}{2}\frac{\delta m}{m_{0}}f_{0}$$

where $f_{0}$ is the resonance frequency of the bare material and $m_{0}$ the initial mass of the sensor.

The performance of this type of mass sensor is mainly determined by two parameters: the sensitivity of the sensor (S) and the quality factor (Q) [12]. The sensitivity indicates the minimum detectable frequency change when the mass of the sensor increases:

(3) $$S=-\frac{\delta f}{\delta m}$$

**Figure 1:**
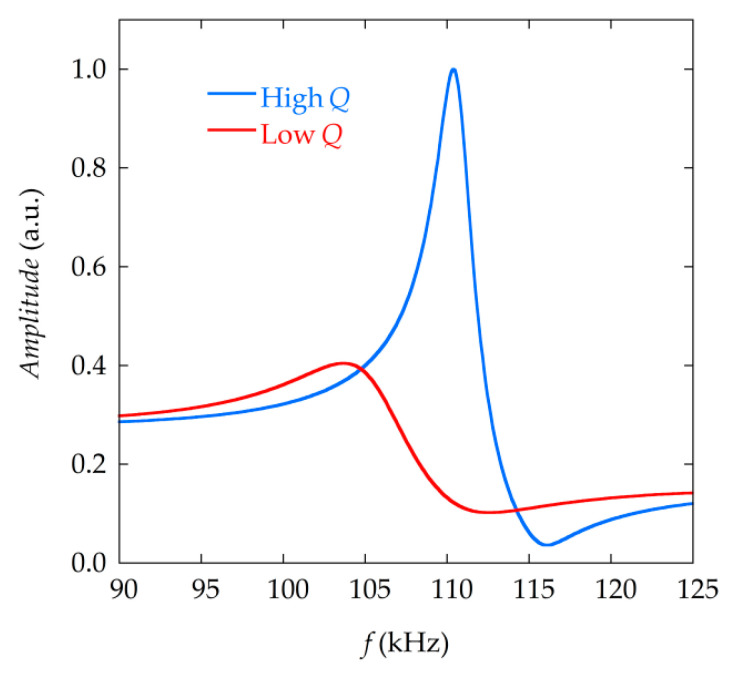
Two magnetoelastic resonance curves from the same resonator with different Q values, caused by different amount of mass loading. The peak corresponding to the higher Q (blue) is sharper and narrower, the resonance curve with a lower Q is wider (red).

The quality factor Q is a dimensionless parameter that describes how sharp the resonance curve is, and decreases when the resonator is damped. Damping influences the shape of the magnetoelastic resonance curve and the resonance frequency itself, considered as the frequency of the maximum of the curve. A high Q value indicates a lower rate of energy loss, a sharp magnetoelastic resonance peak (Figure 1) and therefore, good resolution when determining the resonance frequency and frequency shifts. For developing high performance sensors, the highest S and Q are desirable.

The quality factor can be estimated from the resonance curve as the experimentally measured resonance frequency $f_{max}$ (at which the amplitude is maximum, $A_{max}$), divided by the width of the signal Δf (measured as the full width at half maximum, corresponding to amplitudes $A_{max}/\sqrt{2}$) [13]:

(4) $$Q=\frac{f_{max}}{\Delta f}$$

But the accuracy of this estimation method is very low (it can lead to errors up to 20% in the determination of Q) [14], so finding alternative and robust methods to determine this parameter with greater accuracy is of great importance for the study of the performance of these sensors, especially in cases where the curves have considerable noise or damping, as it is the case when the magnetoelastic sensor operates immersed in a fluid. Some studies have been made on the different methods of determining this quality factor Q [13,15], and Lopes et al. [16] reported an extensive study of the determination of the quality factor of a magnetoelastic sensor using different strategies. In this last work, a numerical fitting of the experimental data to an expression of the magnetic susceptibility of the sample around the resonance is carried out and used as a suitable technique to calculate the value of Q.

In the present work, the numerical fittings of the resonance curves obtained with a magnetoelastic sensor have been carried out using two different expressions: on the one hand, the expression used by Lopes et al. [16], that appears as Equation (6) below in this work and, on the other hand, a phenomenological approach that describes the frequency response of these magnetoelastic sensors and that has already been used to fit these resonance curves [17,18], appearing as Equation (9) within this work. The performance of each of the fitting expressions to determine the quality factor Q has been studied, and their comparison and equivalence carried out.

The resonance curves used in the fit analysis we will show in the following, correspond to those obtained in the monitoring of the precipitation reaction of calcium oxalate salt crystals (inorganic salts resulting from various metabolic activities in humans) using a magnetoelastic sensor. This monitoring process was first described by Bouropoulos et al. [19], and the measurements obtained in this reaction sensing and used in the present work are explained in detail in a previous publication [20]. Through the numerical fitting of these curves, we have studied the evolution of the different parameters that define the resonance curve as the precipitation reaction progress, which provides a better understanding of the effect that the changes of mass in the sensor and damping have on the resonance signal obtained.

## 2. Materials and Methods

### 2.1. Magnetoelastic Material of the Sensor

The material used as sensor platform is a magnetoelastic ribbon of composition ${\mathrm{Fe}}_{73}{\mathrm{Cr}}_{5}{\mathrm{Si}}_{10}B_{12}$ provided by Vacuumschmelze GmbH & Co. KG, Hanau Germany. This material has an excellent corrosion resistance behavior due to the fact that it contains a small amount of chromium (5% at.), which forms a superficial passivation layer in the material. Magnetic, magnetoelastic and corrosion resistance properties of this material were studied by Sagasti et al. [21], and are summarized in Table 1. The strip was laser cut with dimensions 20 mm ×2 mm×25 μm (length to width ratio R=10).

### 2.2. Precipitation Reaction Measurements

The measurements used in this work to make the numerical fittings correspond to the monitoring of the precipitation reaction of calcium oxalate crystals $\left({\mathrm{CaC}}_{2}O_{4}\right)$:

(5) $${\mathrm{CaCl}}_{2}\left(aq\right)+H_{2}C_{2}O_{4}\left(aq\right)\overset{}{\to }{\;\mathrm{CaC}}_{2}O_{4}\left(s\right)+2\mathrm{HCl}\left(aq\right)$$

The formation of the salt crystals during the precipitation process was tracked by detecting changes in the sensor resonance frequency $f_{max}$, as fully described by Sisniega et al. [20]. The magnetoelastic sensor described in the previous subsection was placed in a vial with a mixture of equal parts of the reagents (${\mathrm{CaCl}}_{2}$ and $H_{2}C_{2}O_{4}$) at the same concentration (for three different concentrations: 30, 50, and 100 mM) and the changes in the resonance frequency of the sensor (determined by Equation (2)) were monitored along time, as the crystals precipitate and settle onto the sensor (increasing its mass). The experimental setup used to register the resonance curves consists of three coaxial solenoids: one to apply a constant bias field; a second one to produce the alternating field to magnetostrictively excite the sample; and the third one, consisting of a compensated pick-up coil, to detect the induced magnetization oscillations on the sensor. A spectrum analyzer (HP 3589A, Hewlett-Packard, Palo Alto, CA, USA) working in swept mode is used to produce the excitation and to receive the signal induced in the pick-up coil. After recording the resonance curves, the frequency of the maximum $f_{max}$ and its amplitude are measured using the built-in analysis procedures of the analyzer and transmitted to a control computer. The sweep speed was set looking for a compromise between good curve resolution and the necessary speed to be able to record the whole resonance-antiresonance curve fast enough to follow the precipitation process. With this configuration, the experimental uncertainty in the frequency determination is 100 Hz, and the measurement noise can be up to 1 kHz when the resonance curves are wide and with low amplitude.

**Figure 2:**
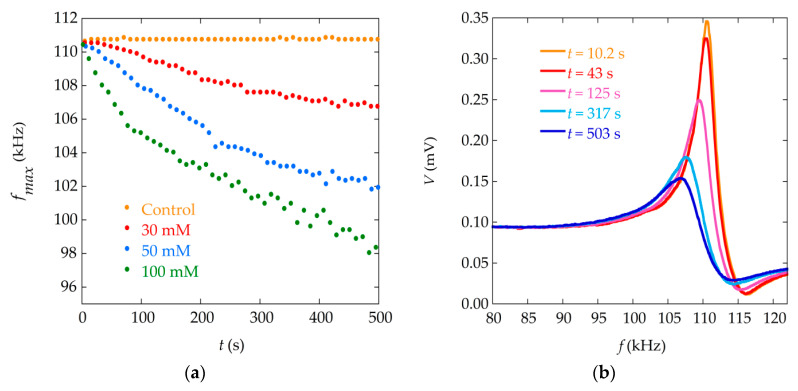
(**a**) Temporal evolution of the magnetoelastic resonance frequency of the sensor during the reaction of precipitation for different concentrations (30 mM, 50 mM and 100 mM) and a control curve (sensor in a vial with distilled water). The value of the resonance frequency, $f_{max}$, is obtained as the frequency at the maximum amplitude of measured resonance curves (Figure 2b); (**b**) Measured magnetoelastic resonance curve signals of the sensor at different times during the precipitation process for the concentration 30 mM. Data taken from Ref. [20].

Figure 2 presents an example of the results obtained in the precipitation monitoring measurements. The curves in Figure 2a show the decrease of the sensor resonance frequency ($f_{max}$) as the precipitation takes place, for the three solutions with different concentrations. Figure 2b shows, for one particular solution (30 mM), the evolution of the complete resonance curve as the reaction progresses (curves taken at different reaction times). It can be seen that, in addition to the decrease in the resonance frequency, there is also a reduction of the quality factor Q that characterizes the resonance (or increase in damping), and a decrease in the amplitude of the sensor signal. The resonance curves displayed in Figure 2b were used in the present work to carry out the numerical fittings, together with analogous curves corresponding to the solutions of concentration 50 and 100 mM. Successive experiments in the same conditions yield similar results. Here we present the analysis in a single run for each concentration. Nine resonance curves corresponding to different reaction times were fitted for each concentration.

### 2.3. Numerial Fitting of the Sensor Resonance Curves

The quality factor Q of the resonance can be quantified from the width and the peak position of the resonance curve. However, when the resonance signals are heavily damped this and other simple methods are unable to determine this parameter or are highly inaccurate. We propose to use a frequency response fitting method to determine the quality factor and other parameters that characterize the resonance curves. In 1978, Savage et al. [22] derived an expression for the susceptibility of the magnetoelastic resonator around the magnetoelastic resonance given by

(6) $$\chi \left(\omega \right)=\chi_{0}\left[1-\frac{8k^{2}}{\pi^{2}}\underset{n}{\sum }\frac{1}{n^{2}}\times \frac{1}{1-\frac{\omega_{n}^{2}}{\omega^{2}}+jQ^{-1}\frac{\omega_{n}}{\omega }}\right]$$

where k is the magnetoelastic coupling coefficient ($k=\sqrt{\frac{\pi^{2}}{8}\left(1-{\left(\frac{f_{r}}{f_{a}}\right)}^{2}\right)}$, $f_{r}$ and $f_{a}$ being the resonance and anti-resonance frequencies, respectively), $\omega_{n}=2\pi f_{n}$ is the resonance frequency of the *n*th harmonic of the excited fundamental mode ($n=1,\;f_{1}=f_{r}$), $Q^{-1}$ is a phenomenological damping coefficient, $\chi_{0}$ is the magnetic susceptibility measured at a frequency far below the resonance and *j*$=\sqrt{-1}$. Due to the experimental procedure used to measure the resonance curves [20], the voltage induced in the pick-up coil (which is displayed in Figure 1 and Figure 2b) is proportional to the magnetic susceptibility. The parameters to be fitted in Equation (6) are: $\omega_{r}$, $\omega_{a}$, $\chi_{0}$, and Q. The numerical fitting of the experimental data to the modulus or magnitude of the Equation (6) for its first resonant mode (n=1) was performed using Mathematica^©^ software. The parameters $\omega_{r}$, $\omega_{a}$, and $\chi_{0}$, were allowed to vary around their experimental corresponding values (extracted from the experimental data), until the value of Q that minimizes the norm between the fit and the experimental data was found. The norm (or residual) is defined as:

(7) $$ℛ=\frac{1}{N}\underset{i=1,N}{\sum }{\left(\frac{\chi_{exp,i}-\chi_{fit,i}}{\chi_{max}}\right)}^{2}$$

where $\chi_{max}=\max\left(\chi_{max,exp},\chi_{max,fit}\right)$, N is the number of experimental points (N=401, in our measurements), and 0≤ℛ≤1, meaning values close to 0 that the fitting is good.

Additionally, a phenomenological expression using the formalism of linear systems was developed to describe the frequency response of a magnetoelastic material [17]. The magnetoelastic resonance is characterized by maximum amplitude at the resonance frequency $f_{r}$, and a null minimum at the anti-resonance frequency $f_{a}$. The analytical expression of the transference function (G) that describes a system with such frequency response is composed of a couple of complex conjugated poles in the denominator (describing the resonance) and a couple of complex conjugated zeros in the numerator (describing the anti-resonance):

(8) $$G\left(s\right)=\frac{\omega_{r}^{2}}{\omega_{a}^{2}}\cdot \frac{s^{2}+2\delta_{a}\omega_{a}s+\omega_{a}^{2}}{s^{2}+2\delta_{r}\omega_{r}s+\omega_{r}^{2}}$$

where $\omega_{r}=2\pi f_{r}$ is the resonance frequency, $\omega_{a}=2\pi f_{a}$ the anti-resonance frequency, and $\delta_{r}$ and $\delta_{a}$ are damping parameters. The transfer function is expressed as a Laplace operator with s=jω, where $j=\sqrt{-1}$ and ω is the frequency.

The amplitude of the frequency response of the system, when submitted to harmonic excitation, based on the frequency response described on the transference function is:

(9) $$V\left(\omega \right)=A\cdot \frac{\omega_{r}^{2}}{\omega_{a}^{2}}\left|\frac{\omega^{2}-2j\delta_{a}\omega_{a}\omega -\omega_{a}^{2}}{\omega^{2}-2j\delta_{r}\omega_{r}\omega -\omega_{r}^{2}}\right|+a\omega +b$$

where A accounts for the amplitude and a and b for the background signal of the experiment (assumed to be linear). The parameters to be fitted in the expression are: $\omega_{r}$, $\omega_{a}$, $\delta_{r}$, $\delta_{a}$, A, a and b. Note that in this expression, the quality factor Q does not appear explicitly. Instead, the damping parameters $\delta_{r}$ and $\delta_{a}$ carry the relevant information. The numerical fitting of Equation (9) to the experimental data was carried out using MATLAB^®^ software and a nonlinear least-squares fitting. The parameters $\omega_{r}$, $\omega_{a}$, and *A* to be fitted are initially taken as their values obtained from the experimental curve. All parameters are fitted until those that best fit the experimental curve are found, in this case those that correspond to a local minimum of a function that is a sum of squared residuals (being the residual the difference between the experimental value of the dependent variable and the value predicted by the fitting model).

## 3. Results and Discussion

### 3.1. Numerical Fitting of the Resonance Curves and Resonance Frequency

**Figure 3:**
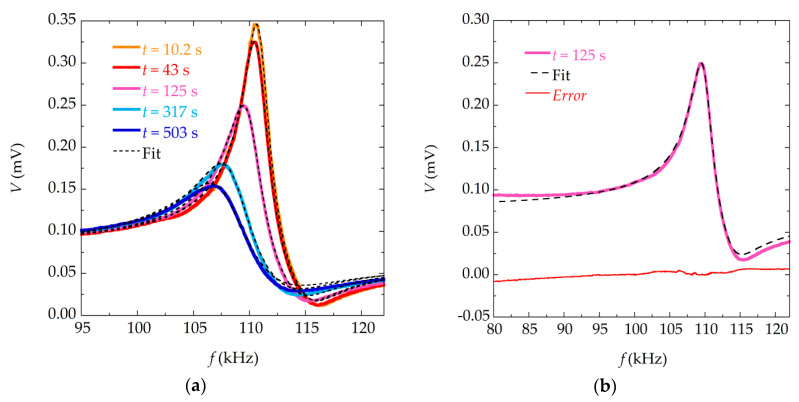
Fit to Equation (6). (**a**) Measured magnetoelastic resonance curves of the sensor at different times during the precipitation process for reactants of concentration 30 mM, and corresponding numerical fittings (in dashed lines) to Equation (6); (**b**) Measured magnetoelastic resonance curve, fitting to Equation (6) (in dashed line), and corresponding error (in red) of the sensor at time *t* = 125 s for reactants of concentration 30 mM.

**Figure 4:**
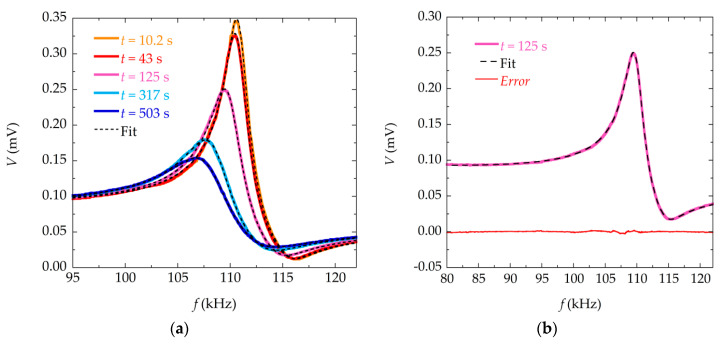
Fit to Equation (9). (**a**) Measured magnetoelastic resonance curves of the sensor at different times during the precipitation process for reactants of concentration 30 mM, and corresponding numerical fittings (in dashed lines) to Equation (9); (**b**) Measured magnetoelastic resonance curve, fitting to Equation (9) (in dashed line), and corresponding error (in red) of the sensor at time *t* = 125 s for reactants of concentration 30 mM.

Numerical fittings were performed to the resonance curves measured at different times in the precipitation reaction, for solution with different concentrations (30, 50 and 100 mM). Figure 3 shows an example of the numerical fittings made with Equation (6), while Figure 4 shows the same fitting but using Equation (9). It can be seen that both models fit the experimental data reasonably well, resulting in a low value of the corresponding residuals. 

However, when the deposited mass is high (at high reaction times or higher concentration of reagents), the fitting to Equation (9) behaves slightly better (with lower error between the experimental data and the values obtained through the model) than the one using the Equation (6).

**Figure 5:**
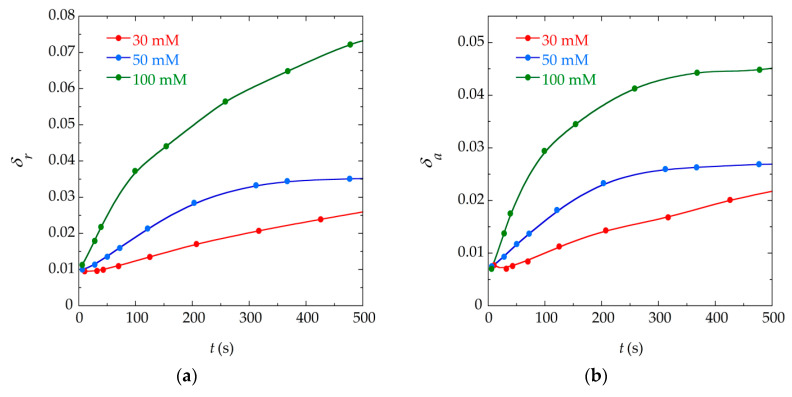
Evolution of the damping parameters obtained through the numerical fitting to Equation (9) of the resonance curves during the precipitation time for different concentration of reagents (30, 50, and 100 mM). (**a**) Damping parameter $\delta_{r}$; (**b**) Damping parameter $\delta_{a}$.

**Figure 6:**
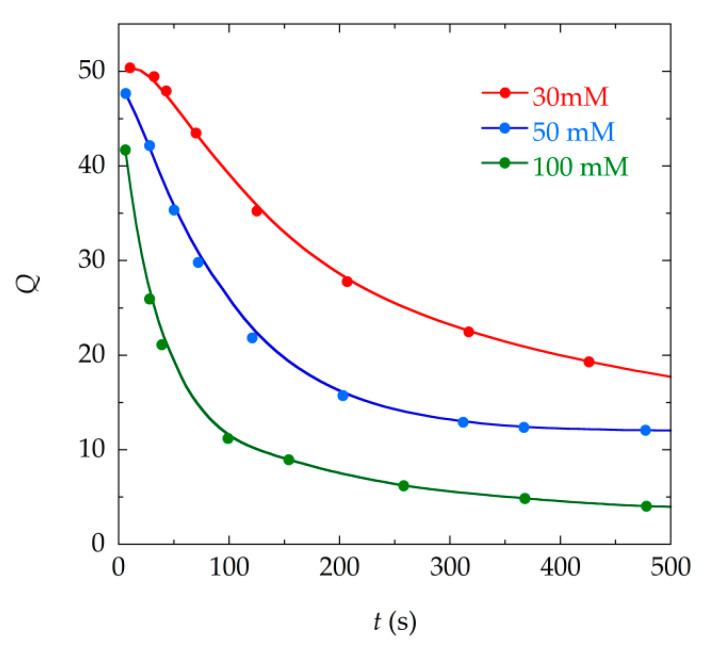
Evolution of the quality factor Q of the resonator during the precipitation process for different reagents concentrations (30, 50, and 100 mM) obtained through the numerical fittings to Equation (6) of the resonance curves.

If we analyze the evolution of the parameters obtained by the fitting procedure, we can see that the two damping parameters in Equation (9) ($\delta_{r}$ and $\delta_{a}$) increase progressively as the mass deposited in the sensor increases (Figure 5). As the reaction advances, the crystals are formed in the solution of the vial and are deposited on the surface of the sensor, increasing its mass, and this increases the damping suffered by this sensor. In the same manner, as the concentration of the reagents increases (30, 50, 100 mM), the mass of calcium oxalate crystals is greater and, therefore, so is the damping. The numerical fittings show this direct relationship between the deposited mass and the damping in the resonance curves obtained. Similarly, it can be seen that the Q parameter obtained by fitting the data to Equation (6) decreases as the precipitation reaction progresses and the mass is deposited on the sensor (Figure 6).

Figure 6 shows the inverse relation between the quality factor Q and the damping parameters ($\delta_{r}$ and $\delta_{a}$). All these parameters show a clear and marked tendency as the precipitation reaction occurs.

**Figure 7:**
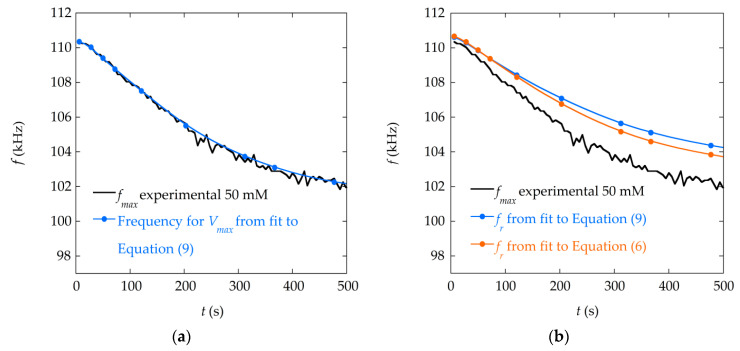
(**a**) Evolution of the frequency corresponding to the maximum of the resonance curves resulting from the fitting to Equation (9) of the resonance curves measured during the precipitation, when the concentration of the reactants is 50 mM, compared to experimental data ($f_{max}$); (**b**) Evolution of the $f_{r}$ obtained in the numerical fitting to Equations (6) and (9) of the measured resonance curves, compared to experimental data ($f_{max}$ ).

The evolution of resonance parameters, preferentially the resonance frequency, can be used to monitor the reaction by the changes caused by the mass deposition on the sensor signal. Numerical fittings improve the information obtained through the changes observed in the measured resonance frequency, since the result comes from the whole resonance curve, not only from the point of maximum amplitude and the width at half maximum. In addition, the fitting procedure is useful to reduce the noise, especially when the signal is low or highly damped, as evidenced in Figure 7a. This plot displays the frequency of the maxima (that is, the resonance frequency $f_{max}$) of all the resonance curves measured during the precipitation process of the solution with concentration 50 mM. The data obtained directly from the measurements present a considerable ripple with an amplitude close to 1 kHz, which severely limits the resolution of the sensor. It is mainly caused by the limited resolution with which frequency differences are discriminated by the measuring system. The evolution of the frequency of the maxima obtained from the fitting of the resonance curves displays a smooth monotonous tendency, which facilitates the calibration and use of the sensor.

One important consequence of the fitting procedure is that the values of the $f_{r}$ parameter obtained in the fittings do not correspond to $f_{max}$, the position of the maxima of the resonance curves. Figure 7b compares the fitted values from both fitting expressions with the experimental resonance frequency ($f_{max}$, taken as the maximum of the resonance curve).

**Figure 8:**
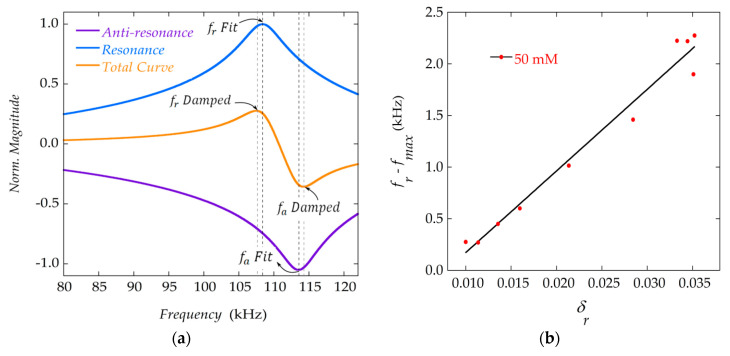
(**a**) Frequency response of the system for resonance and anti-resonance behavior, and the total response affected by the damping. Corresponding resonance and anti-resonance frequencies are indicated; (**b**) Evolution of the difference between the experimental resonance frequency $f_{max}$ and the resonance frequency obtained through the fitting to Equation (9) as the damping parameter $\delta_{r}$ increases, for the case of concentration 50 mM. The shift in the resonance frequency becomes greater as the damping increases.

This tells us that what we usually take as the resonance frequency, that is the frequency of the maximum of the resonance curve, is not really the physical resonance frequency of the sensor, $f_{r}$ which can be obtained from the fitting to the analytical expressions. The frequency of the maximum of the measured curves, $f_{max}$, is systematically lower than the resonance frequency, $f_{r}$, since it is affected by the damping of the curve. The same applies to the frequency of anti-resonance (minimum of the curve), which, when affected by damping due to the mass deposition, increases with respect to the value, $f_{a}$, which is obtained from the fits. Figure 8a illustrates this discrepancy, showing the frequency response of the system, for ideal resonance and anti-resonance separately, and for the full curve (sum of resonance and anti-resonance) affected by damping. In Figure 8b, the difference between the frequency of the maximum of the resonance $f_{max}$ and the resonance frequency $f_{r}$ is shown as a function of the damping, and a linear relation between them is observed.

The curves in Figure 8a were obtained with the magnitude of the frequency response corresponding to the transfer functions representing systems of: a pure resonance ($G_{1}$), a pure anti-resonance ($G_{2}$), and the combination of both ($G_{3}$), which represents the observed resonance-antiresonance behaviour.

(10) $$G_{1}\left(s\right)=\frac{\omega_{r}^{2}}{s^{2}+2\delta_{r}\omega_{r}s+\omega_{r}^{2}}$$

(11) $$G_{2}\left(s\right)=\frac{s^{2}+2\delta_{a}\omega_{a}s+\omega_{a}^{2}}{\omega_{a}^{2}}$$

(12) $$G_{3}\left(s\right)=G_{1}\cdot G_{2}=\frac{\omega_{r}^{2}}{\omega_{a}^{2}}\frac{s^{2}+2\delta_{a}\omega_{a}s+\omega_{a}^{2}}{s^{2}+2\delta_{r}\omega_{r}s+\omega_{r}^{2}}$$

The frequency values obtained for the resonance (maximum value) and anti-resonance (minimum value) of the respective curves in Figure 8a, coincide with those obtained in the fittings ($f_{r}\;Fit$ and $f_{a}\;Fit$ in Figure 8a). In the combined curve, however, the maximum and minimum are shifted with respect to these values, resulting in the values of the resonance and anti-resonance frequencies ($f_{r}\;Damped$ and $f_{a}\;Damped$ in Figure 8a), which match the experimental data.

**Figure 9:**
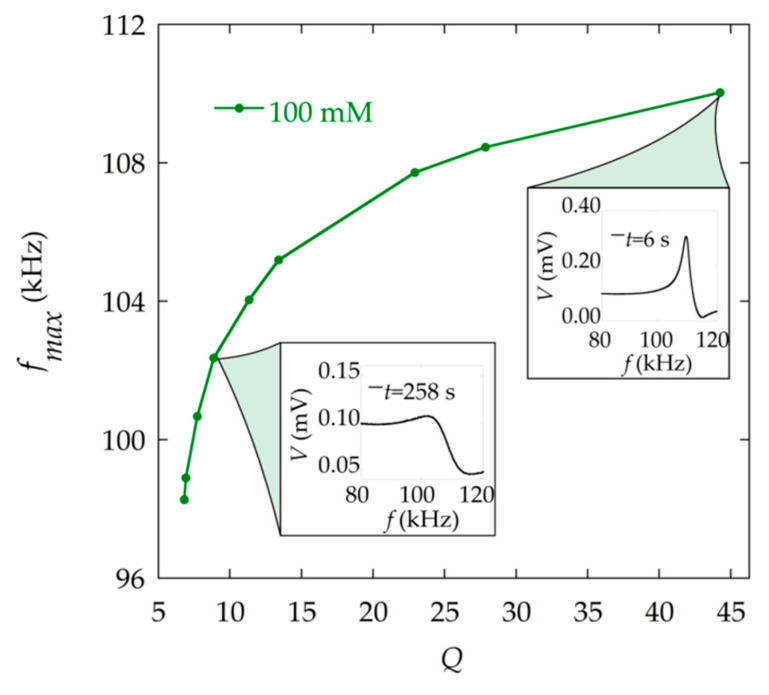
Relation between the resonance frequency $f_{max}$ obtained experimentally and the quality factor Q obtained through the fitting to Equation (9) at different times during the precipitation reaction. The resonance curves corresponding to times *t* = 6 s (high Q ) and *t* = 258 s (low Q ) are shown.

The mass increase on the resonator produces both a decrease of the maximum of the resonance frequency ($f_{max}$) and a decrease of the quality factor. Figure 9 illustrates the correlation between both parameters. However, the observed reduction of $f_{max}$ combines the effect of mass loading (Equation (2)) and the effect described in Figure 8a. The analysis of the resonance curves through the fitting procedure allows the determination of $f_{r}$, which is, in principle, the one that is considered in Equation (2).

### 3.2. Comparison of the Results Obtained with Both Numerical Fittings

As it is to be expected, Equations (6) and (9) are analytically equivalent, when only the first resonant mode (*n* = 1) is considered in Equation (6). By rearranging the Equations (6) and (9) and after simple transformations, the following equivalences between the equation’s parameters are found:

(13) $$\delta_{r}=\frac{1}{2Q}$$

(14) $$\delta_{a}=\frac{\omega_{a}}{\omega_{r}}\frac{1}{2Q}$$

(15) $$A=\chi_{0}$$

**Figure 10:**
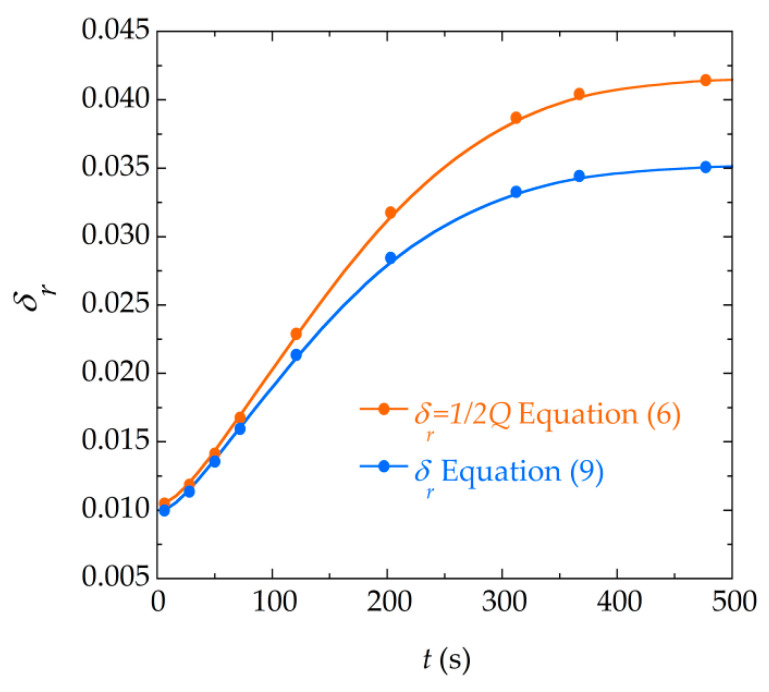
Comparison of the damping parameter $\delta_{r}$ obtained with both numerical fittings of the resonance curves (for reactants of concentration 50 mM) using the equivalences found between $\delta_{r}$ and Q, Equation (13).

**Figure 11:**
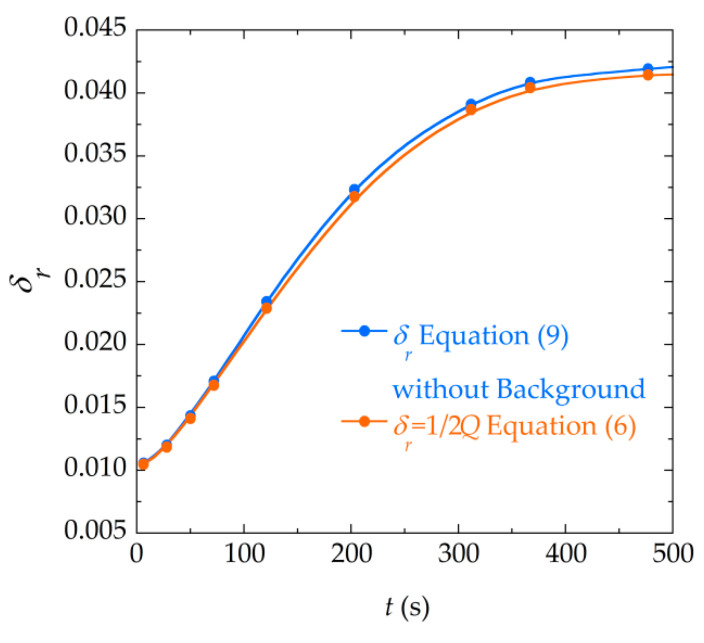
Comparison of the damping parameter $\delta_{r}$ obtained by the fitting to Equation (9) without taking into account the background parameters (a and b ), and the damping parameter $\delta_{r}$ obtained through the fitting to Equation (6).

Figure 10 represents the difference between the fitted parameter $\delta_{r}$ obtained with both numerical fittings, when it is related by means of the above equivalence expressions, for the data corresponding to a precipitation experiment. As can be seen, the difference between the damping parameter increases as the precipitation reaction advances. This is due to the fact that, as the mass of calcium oxalate is deposited in the resonator, the damping increases and the amplitude of the signal becomes smaller. This decrease of the signal causes that the background signal of the measurement gains in importance with respect to the resonance signal. It should be noted that Equation (9) explicitly takes this background into account, unlike Equation (6). For this reason, as the reaction progresses, the effect of the background signal is more noticeable in the fitted parameters. In order to elucidate the role that this background has in the values of the parameters obtained and to be able to compare both expressions under equal conditions (when neither of the two expressions takes this background into account), a fitting to a similar expression to Equation (9) where the background contribution is removed was carried out. The comparison of the damping parameters $\delta_{r}$ obtained with this expression and with Equation (6) is shown in Figure 11. It can be seen that, indeed, in this case both models provide parameters of almost equal value.

Bearing this in mind, we can establish that the background plays an important role to make a proper fitting of the resonance curves of these sensors, especially when working under conditions where the damping is considerable and the signal amplitude is low.

### 3.3. Determination of the Quality Factor

**Figure 12:**
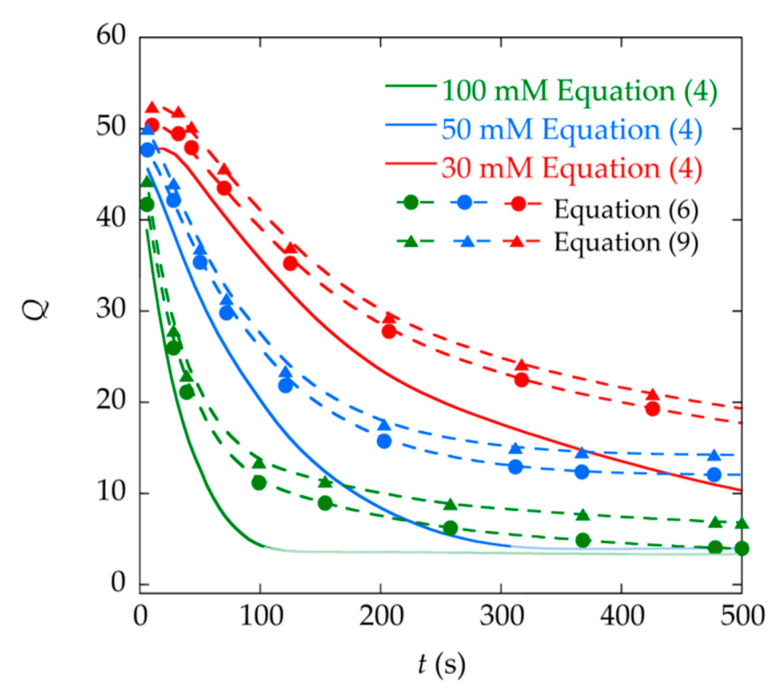
Comparison of the quality factor Q obtained by the fitting to Equations (6) and (9), and the one obtained through Equation (4).

Finally, a comparison of the quality factor obtained by the different procedures was carried out. Figure 12 shows the results of the quality factors obtained, on the one hand by the method of the half maximum bandwidth (Equation (4)), and on the other hand by the numerical fitting, calculating Q from Equation (13) in the case of the fitting to Equation (9). As can be seen, the value of the quality factor Q estimated from Equation (4) is not reliable for these measurements, where the curves are highly damped. In fact, this method is no longer applicable when the bandwidth, Δf, cannot be calculated since the resonance signal becomes small and starts to be buried by the background signal, which occurs for times greater than *t* = 100 s in the case of reagents at a concentration of 100 mM, and for times above *t* = 300 s for concentrations of 50 mM (in continuous line of pale color in Figure 12). Table 2 shows a comparison of the Q values obtained through Equation (4) and through the fitting to Equations (6) and (9), for each concentration of reagents and different reaction times, and relative error between Q obtained through Equation (4) and Equation (9).

**Figure 13:**
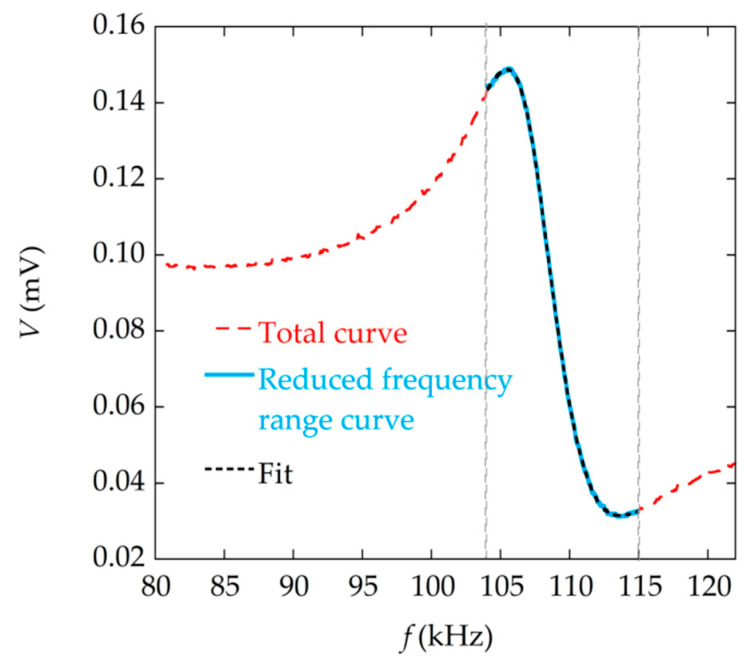
Magnetoelastic resonance curve measured at time *t* = 203 s during the precipitation reaction with 50 mM reactants (in dashed red line), and corresponding fitting to Equation (9) (in dashed black line) for a reduced range of frequencies (in blue). The value of the quality factor obtained with the reduced fitting is Q = 17.4.

In addition, on behalf of a quick measurement, the frequency range in which the resonance curve is measured can be reduced. The fitting procedure can still provide, under these conditions, a valid value of the damping parameter, which the method of the half maximum bandwidth cannot give. As a proof of this, Figure 13 shows the fitting of the resonance curve in a reduced frequency range. The Q value obtained in this way, Q = 17.4, is practically the same as the one obtained with the fitting of the complete curve, in a wider frequency range, Q = 17.5.

Therefore, the quality factors obtained by means of numerical fittings make up for the lack of measurement frequency range and address the difficulty of estimating this parameter when the signals have low amplitude and are highly damped, providing a more accurate determination of Q (as already demonstrated by Lopes et al. [16]). This can be seen in Figure 12 and in data of Table 2, where Q values obtained by the different methods are more similar when the resonance curves are sharper, with less damping (at the beginning of the reaction), and differ as the reaction progresses and the signal quality decreases. In these cases, numerical fitting methods provide a better way to determine the quality factor Q. The result of both expressions (Equations (6) and (9)) used for the fitting is similar, and the slight difference between them arises from considering (or not) the background part (parameters a and b) of the experimental measurements, as shown in Figure 11. Taking this into account, the fitting to Equation (9) is more precise and performs slightly better than the fitting to Equation (6).

## 4. Conclusions

In the present work, we have carried out the numerical fittings of the resonance curves of a magnetoelastic sensor designed to monitor the precipitation reaction of calcium oxalate. Through these fittings, different parameters describing the resonance curves, such as damping parameters, or the quality factor, have been obtained. It has been shown that these parameters are affected by the increase of mass of the sensor when the precipitation reaction advances, and therefore they give us information of how this mass deposition affects the sensor, increasing the damping suffered by it and decreasing its quality factor. These parameters have proven to have potential for monitoring the reaction, as they evolve as the reaction progresses.

The fitting procedure shows that the frequency at which the resonance curve is maximum ($f_{max}$), does not coincide with the resonance frequency of the system, $f_{r}$, as included in the analytical expressions used for the fits. The difference is produced because the damping of the resonance displaces the maximum of the resonance towards lower frequencies.

Clear analytical relationships have been established between the different parameters of the two fitting expressions used. Both expressions have proven to be suitable for making numerical fittings to the resonance curves and provide a very low residual in relation to the experimental data. However, there is a difference between them, and it is mainly due to the adjustment of the background signal, which when taken into account, provides a more precise fitting of the experimental data.

Resonance curve fits have proven to be a great alternative for determining the quality factor Q, and have shown good performance, especially when the resonance curves are highly damped or have low amplitude, where other determination methods have proven to be inaccurate.

## Figures and Tables

**Table 1:** Magnetic, magnetoelastic, and corrosion behaviour parameters of the ${\mathrm{Fe}}_{73}{\mathrm{Cr}}_{5}{\mathrm{Si}}_{10}B_{12}$ sample [21]. $M_{s}$ is the spontaneous magnetization, $\lambda_{s}$ the saturation magnetostriction, ΔE the change in the Young’s modulus with the applied magnetic field (or ΔE effect), k the magnetoelastic coupling coefficient and $E_{corr}$ the corrosion potential.

Composition	$\mu_{0}M_{s}\;\left(T\right)$	$\lambda_{s}$ (ppm)	ΔE (%)	k	$E_{corr}$ (mV)	Corrosion Rate (μm/Year)
Fe_73_Cr_5_Si_10_B_12_	1.12	14	17	0.41	47	0.035

**Table 2:** Comparison of the Q values obtained through Equation (4) and the fitting to Equations (6) and (9) for each concentration of reactants and different times of reaction; and relative error between Q obtained through Equation (4) and Equation (9).

Concentration (mM)	Time (s)	*Q* (Equation (4))	*Q* (Equation (6))	*Q* (Equation (9))	Relative Error * (%)
30	10.2	47.8	50.4	52.4	8.7
	43	45.8	47.9	50.2	8.7
	70	40.5	43.5	45.6	11.2
	125	31.5	35.2	37.0	14.8
	317	16.8	22.5	24.1	30.3
	426	12.6	19.3	20.9	39.7
50	6.3	45.6	47.7	50.0	8.8
	28	38.9	42.1	44.0	11.6
	72	24.6	29.8	31.3	21.4
	121	15.5	21.8	23.4	33.7
	312	-	12.9	15.0	-
	477	-	12.1	14.2	-
100	6	38.9	41.7	44.2	12
	39	14.6	21.1	22.9	36.2
	99	3.6	11.2	13.4	73.1
	154	-	8.9	11.3	-
	368	-	4.9	7.7	-
	478	-	4.1	6.9	-

* Relative error between Q factors obtained through Equation (4) and through the fitting to Equation (9) calculated as: $100\times \left(Q_{Eq.\;\left(9\right)}-Q_{Eq.\left(4\right)}\right)/Q_{Eq.\left(9\right)}$. The cells marked with a dash correspond to the cases where Equation (4) is not applicable.
